# EPHA2, EPHA4, and EPHA6 Expression in Uveal Melanomas: Searching for the Culprits of Neoplasia

**DOI:** 10.3390/diagnostics12051025

**Published:** 2022-04-19

**Authors:** Alexandros Pergaris, Eugene Danas, Pawel Gajdzis, Georgia Levidou, Malgorzata Gajdzis, Nathalie Cassoux, Sophie Gardrat, Piotr Donizy, Penelope Korkolopoulou, Nikolaos Kavantzas, Jerzy Klijanienko, Stamatios Theocharis

**Affiliations:** 1 First Department of Pathology, Medical School, National and Kapodistrian University of Athens, 75 Mikras Asias Street, Bld. 10, Goudi, 11527 Athens, Greece; alexperg@yahoo.com (A.P.); eugenedanas@gmail.com (E.D.); georgia.levidou@klinikum-nuernberg.de (G.L.); pkorkol@med.uoa.gr (P.K.); nkavantz@med.uoa.gr (N.K.); 2 Department of Clinical and Experimental Pathology, Division of Clinical Pathology, Wroclaw Medical University, 50-556 Wroclaw, Poland; pawel.gajdzis@umed.wroc.pl (P.G.); piotrdonizy@wp.pl (P.D.); 3 Department of Pathology, Paracelsus Medical University, 90419 Nuremberg, Germany; 4 Department of Ophthalmology, Wroclaw Medical University, 50-556 Wroclaw, Poland; mgajdzis@usk.wroc.pl; 5 Department of Ophthalmology, Institut Curie, 75005 Paris, France; nathalie.cassoux@curie.net; 6 Department of Biopathology, Institut Curie, PSL Research University, 75005 Paris, France; sophie.gardrat@curie.net; 7 Department of Pathology, Institut Curie, 75005 Paris, France; jerzy.klijanienko@curie.fr

**Keywords:** uveal melanomas, cancer, ephrins, biomarkers, prognosis

## Abstract

Uveal melanomas (UMs) comprise the most common primary intraocular malignancies in adults, with the eye representing the second most common site for melanoma, following the skin. Prognosis remains poor, with approximately half of the cases presenting with metastatic disease at the time of diagnosis. Erythropoietin-producing human hepatocellular receptors (EPHs) comprise the largest known family of tyrosine receptors, in which, along with their ligands, ephrins, play an important role in a plethora of processes in human physiology, and are implicated in key steps of carcinogenesis. In the present study, EPHA2, EPHA4, and EPHA6 immunohistochemical expressions were investigated in UM tissues and further correlated to a multitude of clinicopathological parameters, including disease stage and patients’ overall survival (OS). High levels of EPHA2 expression were significantly associated with increased tumor vertical thickness (*p* = 0.03) and the presence of intrascleral involvement (*p* = 0.05), whereas high EPHA6 nuclear expression was associated with older age at diagnosis (*p* = 0.03) and absence of retinal detachment (*p* = 0.05). In a multivariate survival analysis, increased EPHA4 expression was associated with shortened OS along with the presence of metastasis (*p* < 0.001) and monosomy 3 (*p* = 0.02). In a separate model, the concurrent overexpression of at least two of the investigated EPHs (HR = 14.7, *p* = 0.03) also proved to be an independent poor prognostic factor. In conclusion, our results implicate these specific members of the EPHA group as potential biomarkers for disease prognosis as well as possible targets for the development of novel therapeutic interventions.

## 1. Introduction

### 1.1. Uveal Melanomas

Melanoma can occur anywhere in the body where melanocytes exist, including the uveal tract. The eye is the second most common site for melanoma to develop, following the skin, representing approximately 3% of all melanoma cases [1]. Uveal melanoma (UM) is the most common primary intraocular malignancy in adults, with its incidence amounting to 5.1 cases per million per year [2]. Risk factors for the development of uveal melanoma include, among others, the presence of a Nevus of Ota, nevi of the uvea, fair eyes and skin color, as well as UV radiation [3,4,5,6,7]. About half of the cases present with metastatic disease at the time of diagnosis. Prognosis for this group of patients remains grim, with death rate amounting to 80% at 1 year and 92% at 2 years. Moreover, metastasis can occur years after treatment, regardless of the initial therapeutic strategy utilized [8,9].

A number of clinicopathological parameters have been reported to impact UM patients’ prognoses, as they have been linked to aggressive tumor behaviors and increased rates of metastases. Tumor thickness and inactivating mutations of BAP-1 have been associated with increased risk of metastases [10,11,12] and chromosome 3 monosomy is strongly linked to the presence of metastases as well as decreased survival [13,14,15].

### 1.2. The EPH/Ephrin System

Erythropoietin-producing human hepatocellular receptors (EPHs) comprise the largest known family of tyrosine receptors. Along with their ligands, the EPH family receptor interacting proteins (ephrins) are implicated in a multitude of procedures in human physiology [16]. EPHs are cell membrane proteins, consisting of an extracellular receptor-binding region, as well as a transmembrane and a cytoplasmic domain. As ephrins also represent membrane-bound proteins, EPH–ephrin interaction requires cell-to-cell interaction. Following activation by a ligand, the intracytoplasmic EPH component initiates its receptor tyrosine kinase (RTK) activity, transmitting the signal in the cell through complicated molecular mechanisms, a procedure termed forward signaling. Among others, GTPases of the Rho and Ras family, focal adhesion kinase (FAK), the pathways of the Janus kinase (JAK)-signal transducer and activator of transcription (STAT), as well as the phosphoinositide 3-kinase (PI3K) are implicated in forward signaling [17]. Interestingly, following EPH–ephrin interaction, a response is also triggered in the cytoplasm of the ephrin-expressing cell, a process called reverse signaling. Proteins that interact with the phosphorylated ephrin ligand include Src Homology 2 (SH2) or the PDZ domain containing proteins, such as Grb4 [17].

Nine type A EPHs (EPHA1 to EPHA8 and EPHA10) that bind five ephrin-A ligands (ephrin-A1 to ephrin-A5), along with five type B EPHs (EPHB1 to EPHB4 and EPHB6) that interact with three ephrin-B ligands (ephrin-B1 to ephrin-B3), are expressed in humans [12,13,14,15,16]. Notably, ephrin ligands show a higher affinity for receptors of the same subgroup, although crosstalk between members of different subgroups is also observed [17].

The EPH/ephrin system is implicated in key processes in human physiology. During embryonic development, they participate, among others, in synapse formation, axon guidance, and cell migration. They are also involved in processes, such as cell adhesion, motility, cell–matrix interactions, lymphangiogenesis, and hypoxia-induced angiogenesis [18,19,20,21,22,23]. As most of these procedures also represent essential steps of carcinogenesis, the scientific community has directed its attention towards elucidating the role of EPHs/ephrins in neoplasia. Cell motility is implicated in deeper infiltration of tissues from malignant cells, as well as lymphatic and blood vessel invasion, which are in turn associated with lymph node (LN) and organ metastases. Moreover, tumors cannot grow beyond a few millimeters in diameter without the sprouting of new blood vessels to supply them with oxygen and nutrients [24]. Therefore, the members of the EPH/ephrin system constitute possible biomarkers for neoplastic disease diagnosis, prognosis, as well as targets for the development of novel, personalized therapeutic interventions. Our research team has recently conducted a review of the literature regarding the role of the EPH/ephrin system in solid tumors [25], as well as research proving the utility of these biomolecules as prognostic indicators and therapeutic targets in thymic epithelial tumors [26].

The information regarding the immunohistochemical expression of type A EPHs in UMs is limited to a study investigating EPHA1, EPHA5, and EPHA7 [27]. In the present study, we investigated the immunohistochemical expression of EPHA2, EPHA4, and EPHA6 in UM tissues, associating it with a multitude of clinicopathological characteristics as well as with overall survival (OS) of patients, in an effort to further explore the role of these EPHAs as prognostic biomarkers as well as future therapeutic targets.

## 2. Materials and Methods

### 2.1. Immunohistochemistry

UM formalin fixed paraffin embedded (FFPE) tissue sections were stained with antibodies against EPHA2 (clone ab123877, AbCam/at dilution 1:100); EPHA4 (clone D-4, Santa Cruz, CA, USA/at dilution 1:200), and EPHA6 (clone ab11329, AbCam/at dilution 1:250). Appropriate positive controls with known EPH expression were used as previously described [26,28,29,30]. As negative controls, the omitted primary antibody and substitution with an irrelevant antiserum were used.

Evaluation of immunohistochemistry (IHC) was conducted by two experienced pathologists (S.T. and J.K.) who were blinded to clinicopathological information with complete interobserver compliance. Nuclear and cytoplasmic immunoreactivity was evaluated separately. The extent of nuclear EPHA2, EPHA4, and EPHA6 expression was calculated as the percentage of positive tumor cells to the total number of tumor cells within each section and categorized further in four groups: 0 (no positive cells), 1 (<10% of positive cells), 2 (11–50% positive cells), 3 (51–80% positive cells), and 4 (>80% positive cells). The staining intensity was estimated in four categories: 0 (no reaction), 1 (mild reaction), 2 (moderate reaction), and 3 (intense reaction). An immunoreactive score (IRS) combining percentage of staining multiplied by the staining intensity was created (score 1–12) and then further categorized into four categories: negative expression (IRS 0–1), mild expression (IRS 2–3), moderated expression (IRS 4–8), and strong expression (IRS 9–10). Consequently, negative and mild expressions (IRS 0–3) were categorized into a low expression group, whereas moderate and strong expressions (IRS 4–12) were categorized into a high expression group.

### 2.2. Statistical Analysis

Statistical analysis was performed by a MSc Biostatistician (G.L.). The associations between EPH expression and clinicopathological characteristics were examined using non-parametric tests with correction for multiple comparisons, as appropriate. Survival analysis was performed using Kaplan–Meier survival curves and the differences between the curves were compared with the log-rank test. Numerical parameters were categorized according to their median value. Stepwise-forward Cox regression analysis was performed to evaluate the potential prognostic value of each parameter independent of the remaining parameters. A *p*-value of <0.05 was considered statistically significant. The analysis was performed using the statistical package STATA 11.0/SE for Windows.

## 3. Results

### 3.1. Study Population

Patients’ demographic characteristics are presented in Table 1; 17 patients were men (39%) and 27 were women (61%). The median age at diagnosis was 66.5 years with a range of 14 to 90 years. Tumor thickness varied from 6 to 16 mm with a median value of 12 mm, and basal tumor diameter was from 17 to 24 mm with a median value of 16 mm. There were ciliary body and choroid UM cases, four of which (9%) showed secondary involvement of the iris. Cell type was categorized according to the modified Callender classification system, as follows: 12 epithelioid cell (27%), 23 mixed-cell (52%), and 9 spindle cell melanomas (21%). The T-category according to AJCC was as follows: T2, 2 cases (5%), T3, 20 cases (45%), and T4, 22 cases (50%). Seventeen patients (39%) had metastatic disease at diagnosis. In 19 of the examined 29 cases, a monosomy 3 were documented (66%) and a gain of 8q was present in 13/17 cases (77%). Twenty-four patients (55%) died of their disease within a period of 3 to 146 months. The remaining 20 patients were followed-up for a median period of 66.5 months.

### 3.2. EPHA2 IHC Expression and Association with Clinicopathological Parameters

**Figure 1:**
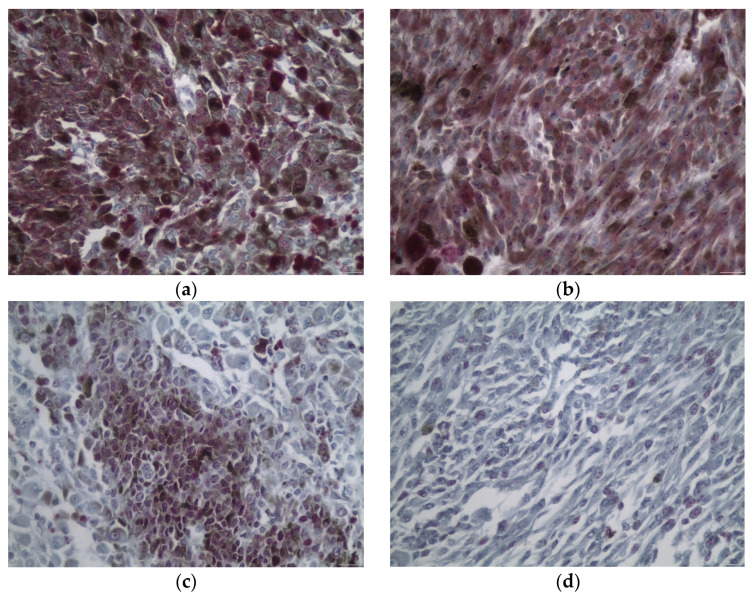
Cytoplasmic and a nuclear IHC staining pattern of EPHA2 (**a**), EPHA4 (**b**), and EPHA6 (**c**), as well as a characteristic case of a nuclear EPHA6 IHC staining pattern (**d**) (×200). (**a**) depicts the only case that exhibited both cytoplasmic and nuclear EPHA2 expression, with the expression pattern in the rest of the cases being only cytoplasmic.

EPHA2 nuclear and cytoplasmic expressions were present in 34 of the cases (77%) (Figure 1). Of the 34 cases that showed EPHA2 expression, 33 cases exhibited a cytoplasmic only staining pattern, with only 1 case showing both nuclear and cytoplasmic EPHA2 expression. Therefore, we proceeded to associate only cytoplasmic EPHA2 expression with clinicopathological parameters. A total of 12 cases (27%) were classified into the high IRS group and 32 cases (73%) into the low IRS group.

**Figure 2:**
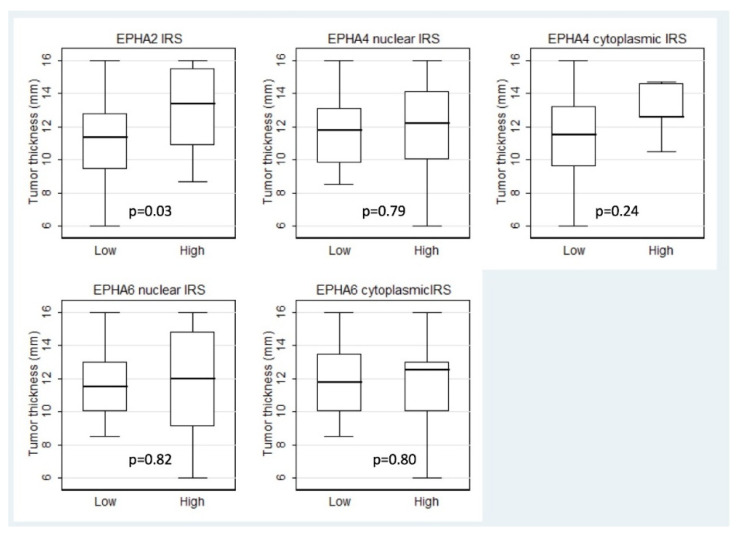
Associations of tumor thickness with EPHA2, EPHA4, and EPHA6 IRS. The low IRS group includes the cases that exhibited completely negative staining.

High EPHA2 IRS was correlated with increased tumor vertical thickness (Mann–Whitney U test, *p* = 0.03, Figure 2), the presence of intrascleral involvement (Fischer’s exact test, *p* = 0.05) and the presence of metastatic disease (Fischer’s exact test, *p* = 0.09), the latter correlation, however, being of borderline significance. The associations with the remaining clinicopathological parameters were not significant (Table 2).

### 3.3. EPHA4 IHC Expression and Association with Clinicopathological Parameters

Nuclear EPHA4 expression was observed in 27 cases (61%), 9 of which displayed a stark staining intensity (Figure 1). Cytoplasmic EPHA4 expression was also documented in 27 cases, with only 5 cases showing a stark staining intensity. Regarding nuclear staining, 32 cases (73%) were classified into the low IRS group and 12 cases (27%) into the high IRS group. In the same context, 39 cases (89%) were classified into the low IRS group for cytoplasmic immunoreactivity and only 5 cases into the high IRS group (11%).

There was no significant association between EphA4 expression and clinicopathological characteristics (Table 3).

The status of cytoplasmic and nuclear IRS in each individual case is presented in Supplementary Figure S1. Concurrent high nuclear and cytoplasmic IRS was observed in only 1 case; 28 cases showed simultaneously low nuclear and cytoplasmic IRS, whereas there was no significant correlation between cytoplasmic and nuclear EPHA4 IRS (Fischer’s exact test, *p* < 0.10).

### 3.4. EPHA6 IHC Expression and Association with Clinicopathological Parameters

Nuclear EPHA6 expression was observed in 28 cases (64%) and cytoplasmic in 22 cases (44%). A total of 29 cases (66%) were classified into the low IRS group and 15 (34%) into the high IRS group according to the nuclear immunoexpression. Accordingly, with regards to the cytoplasmic expression, 30 of cases (68%) were classified into the low IRS group and 14 into the high IRS group (32%) (Figure 1).

High nuclear EPHA6 IRS was correlated with older age at diagnosis (Mann–Whitney U test, *p* = 0.03) and the absence of retinal detachment (Fischer’s exact test, *p* = 0.05). The associations with the remaining clinicopathological parameters were not significant (Table 4).

The status of cytoplasmic and nuclear IRS in each individual case is presented in Supplementary Figure S2. Concurrent high nuclear and cytoplasmic IRS were observed in 4 cases; 19 cases showed simultaneously low nuclear and cytoplasmic IRS, whereas there was no significant correlation between cytoplasmic and nuclear EPHA6 IRS (Fischer’s exact test, *p* < 0.10).

### 3.5. Concurrent Overexpression of EPH2, EPH4, and EPH6

In order to evaluate the role of the concurrent overexpression of the three investigated EPHs, we used the predominant pattern of EPHA4 and EPHA6 immunoreactivity, namely the nuclear expression. In our cohort, there were 15 (34.1%) cases showing overexpression of only one EPH, 9 cases (20.5%) showing concurrent overexpression of two EPHs, and 3 cases (4.5%) showing concurrently high EPH IRS in all three investigated EPHs (Supplementary Figure S3).

The presence of a concurrent expression of the investigated EPHs (either double or triple) was not correlated with any of the clinicopathological parameters, such as tumor thickness (*p* > 0.10).

### 3.6. Survival Analysis

**Figure 3:**
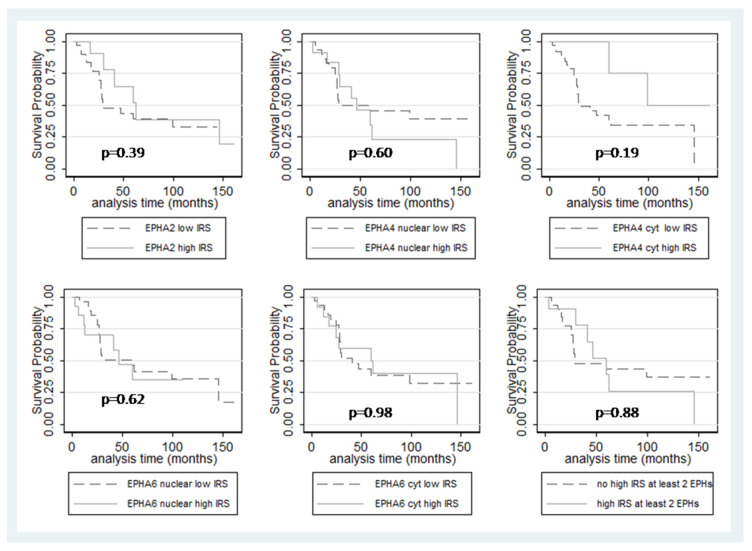
Kaplan–Meier survival curves according to EPHA2, EPHA4, EPHA6 IRS, as well as concurrent overexpression of at least two of the investigated EPHs.

In a univariate survival analysis, monosomy 3 (log-rank test, *p* = 0.03), gain of chromosome 8q (log-rank test, *p* = 0.04), and presence of metastasis (log-rank test, *p* < 0.001) adversely affected OS. Moreover, increased patient age (log-rank test, *p* = 0.06), presence of intrascleral involvement (log-rank test, *p* = 0.06), presence of extrascleral involvement (log-rank test, *p* = 0.08), and increased T-category according to AJCC (T2 vs T3 vs T4, log-rank test, *p* = 0.06) showed an association with adverse outcomes, but these correlations were of marginal significance. EPHA2, EPHA4, und EPHA6 expressions were not informative in this regard (Table 5 and Figure 3). The presence of either single, or concurrent double or triple overexpression of the investigated EPHs, was also not correlated with OS (log-rank test, *p* > 0.10).

In a multivariate survival analysis, we adjusted Cox’s proportional hazard model, including EPHA2 IRS and the predominant pattern of EPHA4 and EPHA6 expression, namely nuclear immunoreactivity, as well as the parameters (presence of metastasis and monosomy 3), which were proven to be significant in the univariate analysis. Chromosome 8q gain was excluded from this analysis due to the small number of cases in which this information was available in our cohort. In this model, high EPHA4 IRS (HR = 7.8, *p* = 0.02) was correlated with a shortened OS along with the presence of metastasis (HR = 30.1, *p* < 0.001) and monosomy 3 (HR = 7.3, *p* = 0.02). Moreover, we adjusted a model, including a parameter representing the concurrent presence of high IRS in at least two of the investigated EPHs along with the presence of metastasis and monosomy 3. In this model, the concurrent overexpression of at least two of the investigated EPHs (HR = 14.7, *p* = 0.03) was proven to be an independent adverse prognosticator, along with the presence of metastasis (HR = 41.9, *p* = 0.02).

## 4. Discussion

UMs constitute a rare type of malignancy, yet the grim prognosis they are usually accompanied with renders the deeper understanding of their biology of great importance. Indeed, scientists have shifted their focus to the discovery of novel biomarkers aiding in early disease diagnosis, prognosis, timely detection of metastasis, as well as molecular candidates that will serve as targets for future, personalized therapeutic strategies. Lamas et al., in a recent review of the literature, presented in a thorough yet concise manner the biomolecules investigated in UM, underlining the progress made in this specific field, with some of the biomolecules bearing a great impact on patients’ prognoses. For instance, loss of nuclear BAP1 staining was linked to an 8-fold higher risk of developing metastases [31]. Malgorzata et al. focused exclusively on biomarkers assessed through IHC, reporting 55 of such molecules that correlate with a multitude of clinicopathological parameters and affecting patients’ outcome [32].

EPHs/ephrins have emerged in the last years as key participants in the process of tumorigenesis. A plethora of studies have proven their link to carcinogenesis, investigating their tumor promoting or tumor suppressing properties [25]. Accordingly, several studies have investigated the immunohistochemical expression of EPHs in a variety of malignancies, often providing conflicting results, a fact suggesting that the role of elevated and/or loss of expression of EPHs is largely context dependent. Certain members of the EPH/ephrin system that seem to suppress carcinogenesis in an organ may enhance neoplasia in another one [25,26,28,29,30]. Moreover, EPHs can be activated independently of the presence of their ligands, by several molecules, such as Ephexin1 and Ephexin4 [33], progranulin [34], and even by EPH–EPH interaction [35]. The respective information regarding the immunohistochemical expression of EPHs in UMs was limited to a recent study performed, which investigated the expression of three type A ephrin receptors, namely EPHA1, EPHA5, and EPHA7 [27]. This study provided promising results regarding the role of these EPHs in Ums; however, the complexity of the EPH/ephrin system remains to be underlined.

In the current study, we aimed to investigate, for the first time, the expression of two of the most often investigated type A EPHs in tumors, namely EPHA2 and EPHA4, along with one of the least investigated, namely EPHA6 in UMs. According to our findings, EPHA2, EPHA4, and EPH6 expressions were observed in the majority of the investigated cases (77%, 61%, and 64%, respectively), EPHA2 showing the higher positivity rate. EPHA2 expression in melanoma was already documented in melanoma cells lines [36]. However, EPHA2, EPHA4, and EPHA6 overexpression was not a frequent phenomenon, since most of our cases were classified into the low expressor group. A similar result was previously observed in the immunohistochemical expression of EPHs A1, A5, and A7 in UMs [27].

Interestingly, EPHA2 immunohistochemical expression was associated with tumor vertical thickness and marginally with the presence of metastasis, both parameters indicating an aggressive clinical behavior. Higher EPHA2 expression has been previously associated with an increased probability of metastasis in several types of tumors, such as in non-small lung cell carcinoma (NSCLC), prostate cancer, and gastric adenocarcinoma [37,38,39]. In particular, the transfection of PC3 cells with kinase/deficient mutant forms of EPHA2 has shown a decreased probability of metastasis when compared to PC3 cells with overexpression of native EPHA2 [40]. Moreover, elevated EPHA2 expression has been associated with increased tumor sizes in gastric adenocarcinoma and gliomas [41,42]. It was previously reported that EPHA2 re-expression in B6 murine melanoma cells activates a non-proteolytic invasive program that proceeds through the activation of cytoskeleton motility, conferring a plasticity in tumor invasiveness [43]. EPHA2 possesses a peculiar model of signaling and a canonical and non-canonical pathway of driving oncogenesis, constituting a ligand- and tyrosine kinase-dependent and independent signaling, respectively [44]. In this context, the invasive signals of EPHA2 in some cases of NSCLC has been attributed to G391R mutation and subsequent phosphorylation of two serines within mTOR [45].

On the other hand, EPHA6 immunohistochemical expression in UMs was associated with older age and the absence of retinal detachment. Information regarding the expression and role of EPHA6 in tumors is limited to a few studies, such as breast carcinoma [46], thymic epithelial tumors [26], and prostate cancer [25], where it might be implicated in angiogenesis and vascular invasion. However, the exact mechanisms with which EPHA6 can potentially promote oncogenesis and disease progression remain to be fully elucidated.

Although EPHA4 immunohistochemical expression did not display in our study any significant association with any of the examined clinicopathological parameters, it was the only Eph receptor that was a single factor in the multivariate analysis correlated with a shortened OS. In order to associate our findings with other reported data, we performed a search of the database cBioportal [47], investigating the mRNA expression of all three genes incorporated in our study (EPHA2, EPHA4, and EPHA6). Interestingly, none of the aforementioned mRNA level genes were observed up- or downregulated or exhibited any association with clinicopathological parameters in UM tumors. Moreover, none of the cases studied exhibited any mutations in those three genes. However, increased EPHA4 expression has been associated with an unfavorable prognosis in a few tumor types, such as in gastric adenocarcinomas [25], whereas knocking down EPHA4 expression in pancreatic adenocarcinomas has been associated with decreased proliferating capacity [48]. Similarly, EPHA4 expression has been correlated with increased chemoresistance and radiotherapy failure in colorectal cancer patients [49]. The results of the survival analysis in our study recapitulated some of the parameters that have been proposed as important determinants of clinical outcome in UMs, namely the presence of metastasis, monosomy 3, and gain 8q, supporting the validity of the statistical analysis and denoting that our cohort, although relatively small, is representative.

Interestingly, activation of a single member of the EPH/ephrin system can lead to different or even opposite results, depending on its location. In our study, nuclear expression of EPHA4 was associated with poorer prognosis, while cytoplasmic expression of the aforementioned molecule exhibited a trend towards better patient outcomes. Husa et al. reported a similar phenomenon in their study of EPHB2 expression in breast cancer specimens. They observed an inverse correlation between membranous and cytoplasmic EPHB2 expression. Membranous EPHB2 expression correlated with longer OS. On the other hand, cytoplasmic EPHB2 expression was linked to shorter OS and was positively associated with histological grade and HER2 expression [50].

An important finding emerging from the present investigation is the fact that the concurrent overexpression of at least two of the investigated EPHs correlated with a decreased OS, remaining as an independent prognostic factor in a multivariate survival analysis, along with the presence of metastasis. This result is not without precedence since the combined expressions of various EPHs have been previously used to distinguish various stages of lung carcinoma [29]. It could be hypothesized that EPHs have, in several tumors, a synergistic or complementary role in order to execute their tumor promoting or tumor suppressing functions and the outcome depends on the expression of specific sets of EPHs. It is well known that EPHs play a complex role in oncogenesis, which is reportedly dependent on the specific interactions among the receptors themselves, ligands, signaling pathways, and adaptor proteins [44]. The significant association of the overexpression of EPHs with a shortened overall survival can be attributed to several functions of these molecules, such as their role in regulating stemness in a subpopulation of cancer cells, responsible for resistance to therapy or their ability to regulate epithelial–mesenchymal transition, to control the cell motility and to alternate the AKT and MAP kinase pathways [44]. However, since all of the aforesaid results and clinicopathological correlations stem from a single cohort, further validation of the impact of EPHAs in the pathogenesis of UM is rendered necessary.

In the last few years, the repeatedly established tumor promoting or tumor suppressive role of the various EPH/ephrins members renders them potential targets of therapeutic intervention. Many different approaches have been employed in this effort, such as antibodies, peptides, and soluble fragments of the receptors/ligands or small inhibitors of protein–protein interactions, kinase inhibitors, and siRNAs [51]. Specifically, a variety of monoclonal antibodies have been tested on several solid tumors or hematologic malignancies, providing promising results, whereas research has been conducted towards a creation of a protein with the ability to block EPH/ephrin interactions [19]. However, since the function of the EPH/ephrin complex is largely executed in a context-specific manner, the understanding of the exact role of each member seems to be of greater importance. Our study, in accordance with our previous investigation on EPHA1, A5, and EPHA7 [27], provides evidence supporting the role of the EPH/ephrin system in UM carcinogenesis. However, it is obvious that further research on the subject is needed to elucidate the exact nature of the various EPHs/ephrins in UM tumorigenesis as well as their potential as future targets of therapeutic interventions.

## Figures and Tables

**Table 1:** Clinicopathological characteristics of 44 patients with UM.

Parameter	Median	Range
**Age**	66.5	14–90 years
**Number of Mitoses per 40 HPFs**	3	0–24
**Vertical thickness**	12	6–16 mm
**Basal diameter**	16	17–24 mm
	**Number**	**%**
**Gender**		
Male	17/44	39%
Female	27/44	61%
**Posterior pole involvement**	15/44	34%
**Ciliary body involvement**	12/44	27%
**Secondary iris involvement**	4/44	9%
**Iridocorneal angle involvement**	3/44	7%
**Presence of retinal detachment**	18/44	41%
**Presence of vitreous hemorrhage**	9/44	20%
**Intrascleral involvement**	35/44	80%
**Extrascleral involvement**	6/44	14%
**Histological cell type**		
Epithelioid cell	12/44	27%
Mixed cell	23/44	52%
Spindle cell	9/44	21%
**Loss of chromosome 3**	19/29	66%
**Gain 8q**	13/17	77%
**Presence of metastasis**	17/44	39%
**T-category (AJCC)**		
Τ1	0/44	0%
Τ2	2/44	5%
Τ3	20/44	45%
Τ4	22/44	50%
**Event**		
Death of disease	24/44, within 3–146 months	55%
Censored	20/44, follow-up 1–162 months	45%

**Table 2:** Associations of EPHA2 with clinicopathological parameters. Cases that were completely negative for EPHA2 expression were classified in the low IRS group.

	Cytoplasmic EPHA2 Expression		
	Low (IRS 0–3)	High (4–12)	*p*-Value
**Parameter**	Median (range)		
**Age**	66.5 (14–90)	70.5 (32–90)	0.33
**Number of Mitoses per 40 HPFs**	3 (0–24)	3 (0–12)	0.88
**Thickness**	11.35 (6–16)	13.35 (8.7–16)	**0.03**
**Basal diameter**	16 (7–24)	14.5 (14–22)	0.29
	Number of cases		
**Gender**			
Male	13	4	0.74
Female	19	8	
**Posterior pole involvement**			
No	21	8	>0.99
Yes	11	4	
**Ciliary body involvement**			
No	23	9	>0.99
Yes	9	3	
**Secondary iris involvement**			
No	28	12	0.56
Yes	4	9	
**Iridocorneal angle involvement**			
No	29	12	0.55
Yes	3	0	
**Presence of retinal detachment**			
No	17	9	0.30
Yes	15	3	
**Presence of vitreous hemorrhage**			
No	27	8	0.23
Yes	5	4	
**Intrascleral involvement**			
No	9	0	**0.05**
Yes	23	12	
**Extrascleral involvement**			
No	26	12	0.17
Yes	6	0	
**Histological cell type**			
Epithelioid cell	10	2	0.36
Mixed cell	17	6	
Spindle cell	5	4	
**Loss of chromosome 3**			
No	9	1	>0.99
Yes	16	3	
**Gain 8q**			
No	3	1	>0.99
Yes	11	2	
**Presence of metastasis**			
No	15	10	**0.09**
Yes	10	2	
**T-category (AJCC)**			
Τ1	0	0	>0.99
Τ2	2	0	
Τ3	14	6	
Τ4	16	6	

**Table 3:** Associations of nuclear and cytoplasmic EPHA4 with clinicopathological parameters.

	EPHA4 Expression					
	Nuclear			Cytoplasmic		
	**Low (IRS 0–3)**	**High (4–12)**	***p*-Value**	**Low (IRS 0–3)**	**High (4–12)**	***p*-Value**
**Parameter**	**Median (range)**			**Median (range)**		
**Age**	65 (14–90)	75 (15–87)	0.33	67 (14–90)	58 (32–90)	0.84
**Number of Mitoses per 40 HPFs**	3 (0–24)	4.5 (0–24)	0.73	3 (0–24	3 (2–12)	0.79
**Thickness**	11.7 (8.5–16)	12.2 (6–16)	0.79	11.5 (6–16)	12.6 (10.5–14.7)	0.24
**Basal diameter**	16 (7–24)	16 (10–20.5)	0.88	16 (7–24)	15 (12–19.5)	0.40
	**Number of cases**			**Number of cases**		
**Gender**						
Male	13	4	0.74	16	1	0.64
Female	19	8		23	4	
**Posterior pole involvement**						
No	21	8	>0.99	23	3	>0.99
Yes	11	4		13	2	
**Ciliary body involvement**						
No	25	7	0.26	28	4	>0.99
Yes	7	5		11	1	
**Secondary iris involvement**						
No	30	10	0.30	35	5	>0.99
Yes	2	2		4	0	
**Iridocorneal angle involvement**						
No	31	10	0.18	36	5	>0.99
Yes	1	2		3	0	
**Presence of retinal detachment**						
No	19	7	>0.99	24	2	0.39
Yes	3	5		15	3	
**Presence of vitreous hemorrhage**						
No	26	9	0.69	31	4	>0.99
Yes	6	3		8	1	
**Intrascleral involvement**						
No	6	3	0.69	8	1	>0.99
Yes	26	9		31	4	
**Extrascleral involvement**						
No	26	12	0.17	33	5	>0.99
Yes	6	0		6	0	
**Histological cell type**						
Epithelioid cell	10	2	0.60	12	0	0.31
Mixed cell	15	8		20	3	
Spindle cell	5	2		7	2	
**Loss of chromosome 3**						
No	6	4	0.39	8	2	0.27
Yes	15	4		18	1	
**Gain 8q**						
No	3	1	>0.99	2	2	0.12
Yes	9	4		12	1	
**Presence of metastasis**						
No	18	9	0.31	23	4	0.63
Yes	14	3		16	1	
**T-category (AJCC)**						
Τ1	0	0	0.74			
Τ2	1	1		2	0	0.73
Τ3	15	5		17	3	
Τ4	16	6		20	2	

**Table 4:** Associations of nuclear and cytoplasmic EPHA6 with clinicopathological parameters.

	EPHA6 Expression					
	Nuclear			Cytoplasmic		
	**Low (IRS 0–3)**	**High (4–12)**	***p*-Value**	**Low (IRS 0–3)**	**High (4–12)**	***p*-Value**
**Parameter**	**Median (range)**			**Median (range)**		
**Age**	63 (14–90)	75 (15–90)	**0.03**	65 (15–90)	73.5 (14–85)	0.37
**Number of Mitoses per 40 HPFs**	4 (0–10)	2 (0–24)	0.76	3.5 (0–24)	3 (0–24)	0.83
**Thickness**	11.5 (8.5–16)	12 (6–16)	0.82	11.7 (8.5–16)	12.5 (6–16)	0.80
**Basal diameter**	16 (10–24)	16 (7–22)	0.59	16.5 (7–24)	16 (10–20)	0.54
	**Number of cases**			**Number of cases**		
**Gender**						
Male	18	11	0.52	19	10	0.74
Female	11	4		11	4	
**Posterior pole involvement**						
No	18	11	0.52	19	10	0.74
Yes	11	4		11	4	
**Ciliary body involvement**						
No	23	9	0.28	22	10	>0.99
Yes	6	6		8	4	
**Secondary iris involvement**						
No	26	14	>0.99	27	13	>0.99
Yes	3	1		3	1	
**Iridocorneal angle involvement**						
No	28	13	0.26	27	14	0.54
Yes	1	2		3	0	
**Presence of retinal detachment**						
No	14	12	**0.05**	19	7	0.51
Yes	15	3		11	7	
**Presence of vitreous hemorrhage**						
No	23	12	>0.99	24	11	>0.99
Yes	6	3		6	3	
**Intrascleral involvement**						
No	5	4	0.46	6	3	>0.99
Yes	24	11		24	11	
**Extrascleral involvement**						
No	25	13	>0.99	26	12	>0.99
Yes	4	2		4	2	
**Histological cell type**						
Epithelioid cell	10	2	0.31	9	3	0.70
Mixed cell	13	10		16	7	
Spindle cell	6	3		5	4	
**Loss of chromosome 3**						
No	8	2	0.41	6	4	0.70
Yes	11	8		13	6	
**Gain 8q**						
No	3	1	>0.99	1	3	0.25
Yes	10	3		9	4	
**Presence of metastasis**						
No	16	11	0.33	17	10	0.51
Yes	13	4		13	4	
**T-category (AJCC)**						
Τ1	0	0	0.88	0	0	0.56
Τ2	1	1		1	1	
Τ3	14	6		15	5	
Τ4	14	8		14	8	

**Table 5:** Results of univariate survival analysis (log-rank test).

Parameter	*p*-Value
Age (<66.5 vs. ≥66.5 years)	0.06
Number of Mitoses per 40 HPFs (absence vs. presence)	0.60
Vertical thickness (<12 vs. ≥12 mm)	0.49
Basal diameter (<16 vs. ≥16 mm)	0.94
Presence of retinal detachment (no vs. yes)	0.37
Presence of vitreous hemorrhage (no vs. yes)	0.98
Intrascleral involvement (no vs. yes)	0.06
Extrascleral involvement (no vs. yes)	0.08
Histological cell type (epithelioid vs. mixed vs. spindle cell)	0.47
Loss of chromosome 3 (no vs. yes)	**0.03**
Gain 8q (no vs. yes)	**0.04**
Presence of metastasis (no vs. yes)	**<0.001**
T-category (AJCC) (T2 vs. T3 vs. T4)	0.06
Ephrin A2 IRS (low vs. high)	0.40
Ephrin A4 nuclear IRS (low vs. high)	0.60
Ephrin A4 cytoplasmic IRS (low vs. high)	0.19
Ephrin A2 nuclear IRS (low vs. high)	0.62
Ephrin A2 cytoplasmic IRS (low vs. high)	0.98
Concurrent high expression of at least two EPHs (no vs. yes)	0.88

## Data Availability

The data presented in this study are available upon request from the corresponding author.

## Supplementary Materials

The following supporting information can be downloaded at: https://www.mdpi.com/article/10.3390/diagnostics12051025/s1, Figure S1: EPHA4 nuclear and cytoplasmic level of expression in all cases investigated. Figure S2: EPHA6 nuclear and cytoplasmic level of expression in all cases investigated. Figure S3: EPHA2 cytoplasmic as well as EPHA4 and EPHA6 nuclear expression levels in all cases inves-tigated.
