# Supplementary material for: EPHA2, EPHA4, and EPHA6 Expression in Uveal Melanomas: Searching for the Culprits of Neoplasia
Source: Diagnostics (Basel). 2022 Apr 19;12(5):1025. doi: 10.3390/diagnostics12051025 (PMC9139903; doi:10.3390/diagnostics12051025)
Supplement: Supplementary file 1 [file diagnostics-12-01025-s001.zip › diagnostics-1628255-supplementary.pdf]

diagnostics

# EPHA2, EPHA4 and EPHA6 expression in Uveal Melanomas: Searching for the culprits of Neoplasia

**Alexandros Pergaris[1†], Eugene Danas[1†], Pawel Gajdzis[2], Georgia Levidou[1,3], Malgorzata Gajdzis[4], Nathalie Cassoux[5], Sophie Gardrat[6], Piotr Donizy[2], Penelope Korkolopoulou[1], Nikolaos Kavantzas[1], Jerzy Klijanienko[7] and Stamatios Theocharis[1*]**

## Supplementary material

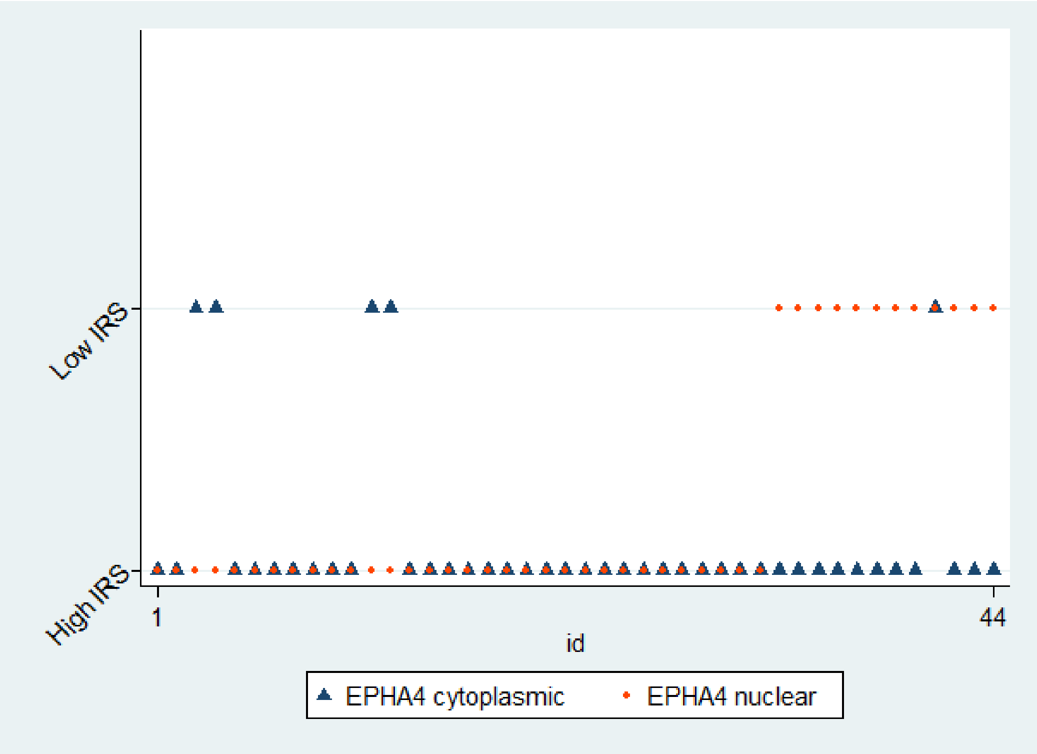


**Figure S1:** EPHA4 nuclear and cytoplasmic level of expression in all cases investigated.

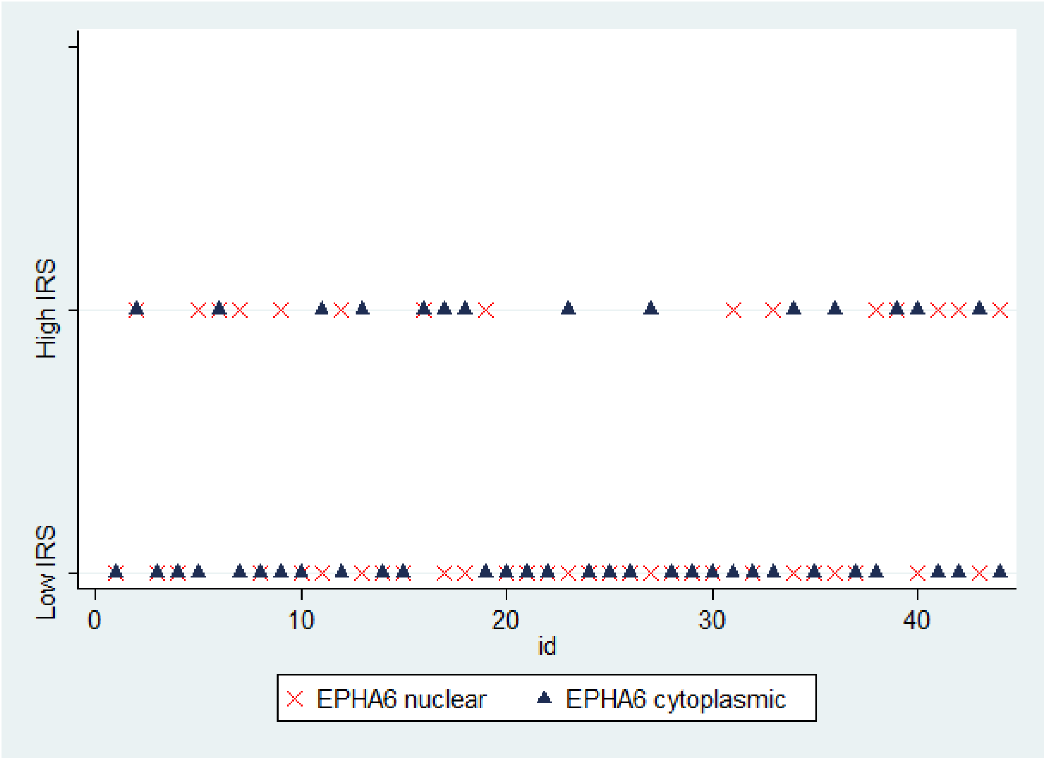


**Figure S2:** EPHA6 nuclear and cytoplasmic level of expression in all cases investigated.

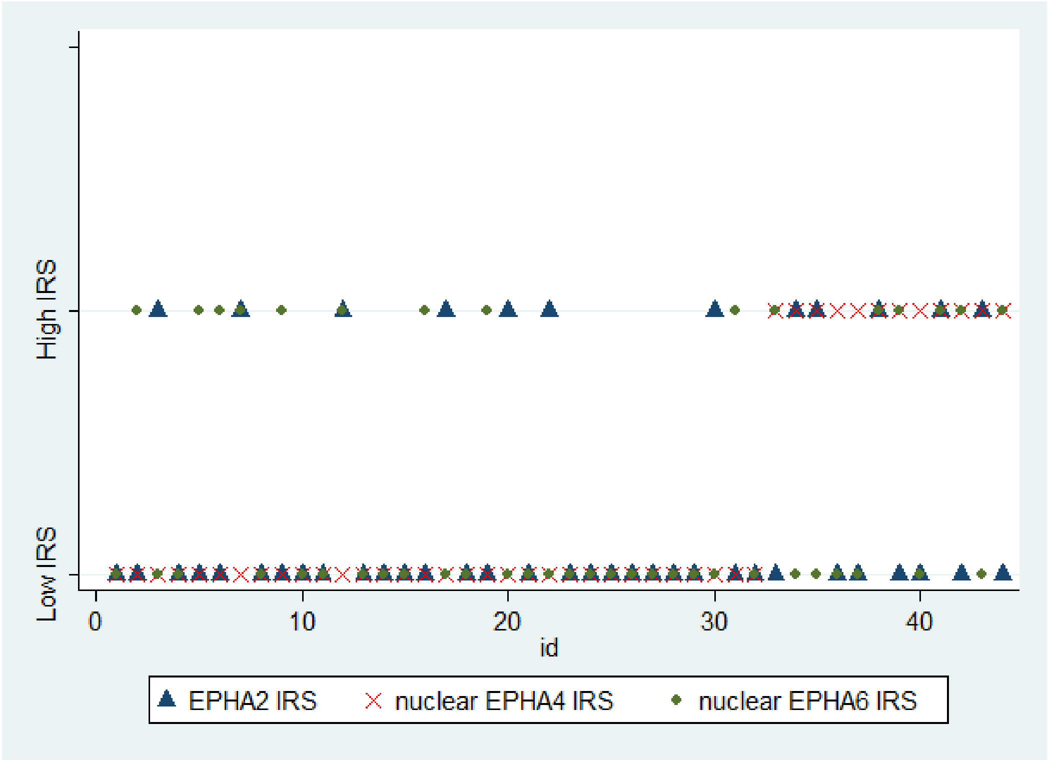


**Figure S3:** EPHA2 cytoplasmic as well as EPHA4 and EPHA6 nuclear expression levels in all cases inves-tigated.
